# Physical Activity in South Asians: An In-Depth Qualitative Study to Explore Motivations and Facilitators

**DOI:** 10.1371/journal.pone.0045333

**Published:** 2012-10-10

**Authors:** Ruth Jepson, Fiona M. Harris, Alison Bowes, Roma Robertson, Ghizala Avan, Aziz Sheikh

**Affiliations:** 1 Centre for Population Health and Public Health Research, School of Nursing, Midwifery and Health, University of Stirling, Stirling, Scotland; 2 Nursing, Midwifery and Allied Health Professions Research Unit, University of Stirling, Stirling, Scotland; 3 School of Applied Social Science, University of Stirling, Stirling, Scotland; 4 Edinburgh Ethnicity and Health Research Group, Centre for Population Health Sciences, The University of Edinburgh, Edinburgh, United Kingdom; Federal University of Rio de Janeiro, Brazil

## Abstract

**Background:**

People of South Asian backgrounds living in the UK have a five-fold increased risk of diabetes and a two-fold increased risk of heart disease when compared to the general population. Physical activity can reduce the risk of premature death from a range of conditions. The aim of the study was to explore the motivating and facilitating factors likely to increase physical activity for South Asian adults and their families, in order to develop successful interventions and services.

**Methodology/Principal Findings:**

This was a qualitative study using focus groups and in-depth interviews. Participants were 59 purposively selected Bangladeshi-, Indian- and Pakistani-origin men and women with an additional 10 key informants. The setting was three urban areas of Scotland: Aberdeen, Glasgow and Edinburgh. We undertook a theoretically informed thematic analysis of data. Study participants described engaging in a range of physical activities, particularly football and the gym for men, and walking and swimming for women. The main motivators for taking part in physical activity were external motivators – i.e. undertaking physical activity as a means to an end, which included the opportunities that physical activity provided for social activity and enjoyment. The goals of weight reduction and improving mental and physical health and were also mentioned. Role models were seen as important to inspire and motivate people to undertake activities that they may otherwise lack confidence in. Few people undertook physical activity for its own sake (intrinsic motivation).

**Conclusions/Significance:**

Attempts at promoting physical activity in people of South Asian origin need to take account of the social context of people's lives and the external motivators that encourage them to engage in physical activity. Undertaking group based physical activity is important and can be facilitated through religious, community, friendship or family networks. Role models may also prove particularly helpful.

## Introduction

People of South Asian origin or descent living in the UK have a substantially increased risk of a range of long-term conditions associated with obesity, including diabetes and cardiovascular disease. In middle-aged UK South Asians with diabetes, for example, both morbidity and mortality from ischaemic heart disease are markedly increased compared to the UK majority population [1], [2], [3]. These risks are particularly pronounced in people of Bangladeshi and Pakistani origin. Unfavourable risk factors for diabetes and heart disease have now also been shown to be present from the school-age years, thus underscoring the need for strategies to reduce risk throughout the lifecourse [4]. While this study is based in Scotland, our findings have wider application to South Asian migrants living elsewhere, given the associated health risks that transcend international boundaries.

South Asia is a term commonly used to denote the geographic area including the countries of India, Nepal, Pakistan, Bangladesh, Bhutan and Sri Lanka. ‘South Asian’ is the term adopted by census, surveys and other government instruments to identify the collective ethnicities of migrants and their descendants from these countries of origin. The 2001 Scottish census showed that South Asians formed 1.09% of the total population of just over 5 million. The highest numbers of South Asians living in Scotland are Pakistani by origin or descent [5]. Furthermore, over 75% of those who identify themselves as ‘Pakistani’ were born in Scotland [6]. Religious observance (predominantly Islam and the Sikh religions in Scotland) and shared languages (particularly Urdu and Punjabi) contribute to a more collective identity although we are aware that these are, of course, cross-cut by other socio-economic differences. Although the term ‘South Asian’ encapsulates a diverse population with varying migration histories, countries of origin, languages and religions, for the purposes of this paper we use this term to refer to a population sub-group that nevertheless share similar health risks and cultural barriers towards physical activity.

Physical activity can reduce the risk of premature death from cardiovascular disease and type 2 diabetes and provide a range of other health benefits [7]. However, South Asians living in the UK have consistently been shown to have lower levels of physical activity than the general population [8], [9], a trend which begins in early life [10], [11], and is likely to be an important contributor to this increased incidence and poor health outcomes. Recent UK research has shown that deaths from coronary heart disease are substantially more common in people from South Asian backgrounds than those of White origins,(hazard ratio: 2.87, 95%CI 1.74 to 4.73) with this increased risk being particularly evident in Bangladeshi- and Pakistani-origin populations [12]. Physical inactivity explained more than 20% of the excess. The authors concluded that “the research highlights the importance of prioritising the promotion of physical activity in this high-risk population” [12].

Several factors distinct to UK South Asians may impact on their physical activity levels. Research indicates that there are significant structural barriers, including a lack of women-only facilities or activity sessions [13]–[16], a lack of confidence among some Muslim women to attend exercise classes [17], and a lack of understanding of the need to integrate physical activity into a normal lifestyle [14]. Furthermore, some studies have demonstrated a lack of understanding in people of South Asian origin regarding the extent of exercise that conferred health benefits. For instance, a study showed that some people believed *namaz* (prayer) was sufficient exercise [17], while another study reported that older people regarded exercise as tiring and that at their age they should “rest or slow down” [15]. However, it is important to note the potential implications of migration histories as South Asians born in the UK report higher levels of physical activity than those born elsewhere [18]. Our research in this area suggests that some of the barriers for UK South Asians are broadly similar to those facing the UK population as a whole (childcare issues, lack of time, and motivation) [19].

There is currently little evidence of successful physical activity interventions amongst South Asian groups. Given the well-described low levels of exercise and the associated persistent structural and other barriers – which have now extended over two or more generations – promoting physical activity in acceptable and sustainable ways for these populations is likely to prove particularly challenging. Although several studies have investigated barriers, no studies have explored in-depth what motivates people from South Asian backgrounds to undertake physical activity, which is likely to be at least equally as important in the development of strategies to promote physical activity.

We were commissioned by NHS Health Scotland to undertake a study to explore the barriers, motivators and facilitators to South Asian adults undertaking physical activity, with the broader aim of guiding the development of future interventions and services [19]. The main aims of the research were to:

1) describe the types of physical activities South Asians residing in the UK engage in; 2) identify motivating and facilitating factors; to provide recommendations for the development of future services.

The findings on barriers largely echoed those reported in the literature previously [13]–[15] so in this paper we focus on the more original contributions relating to motivating and facilitating factors.

## Methods

We undertook a qualitative study employing insights from sociology and anthropology on theories of ethnicity, with sports science and public health perspectives. Using maximum variation sampling, we aimed to include the widest possible range of perspectives, experiences, and views, within focus groups with people of South Asian origin. We used focus groups as our primary data collection method as we considered physical activity to be a non-sensitive subject for discussion and debate. Focus groups were supplemented by semi-structured interviews with key informants, including physical activity specialists/providers and those involved in the delivery of health care or community-based activities to the South Asian population. These additional in-depth interviews with key informants aimed to provide more detailed information and insights on service provision.

### Sampling and recruitment

The geographical locations for the focus groups were the urban centres of Aberdeen, Edinburgh and Glasgow. Building on previous work on the recruitment of ethnic minorities [20]–[22], we adopted a pragmatic, multi-faceted approach to recruitment. We purposively sampled pre-existing or natural groups (e.g. groups who exercised together, mother and baby groups, prayer groups) to reflect the range of countries of origin (Bangladesh, India and Pakistan), gender, socioeconomic status, religious affiliations (Hindu, Muslim and Sikh), and occupational backgrounds that are typical of the South Asian community in Scotland. We actively sought out a range of perspectives (maximum variation), including those that were outliers, to capture as wide a range of perspectives as possible. Rather than using racial and ethnic categorisation, which may be complicit with racial typologies and thinking, we included people within the target age group who defined themselves as being of South Asian origin [23]. We also purposively sampled people who were already involved in physical activities (e.g. football, swimming, keep fit) to identify motivators and facilitators. Table S1 provides further details of the sample characteristics.

We used the postcodes provided by participants to determine the level of affluence or deprivation of their residential area by linking it with the Scottish Index of Multiple Deprivation (SIMD) rankings [24] which were converted to SIMD deciles [25]. This Index identifies small area concentrations of multiple deprivation across Scotland. Participants came from a wide range of socio-economic groups and approximately 12% lived in one of the 10% most deprived areas in Scotland. Within each of the focus groups there was a mix of people from affluent and deprived postcode areas.

As groups were recruited through either existing contacts or ‘gatekeepers’ it is not possible to give exact details of who accepted or declined to participate in focus groups. However, although we used a purposive sampling strategy, this was merged with techniques of theoretical sampling. Therefore, in advance of recruitment we devised target sampling characteristics that would ensure that our sample achieved an appropriate mix of active versus non-active participants as well as participants from a range of socio-economic and religious/cultural backgrounds. One limitation of our sample is that we failed to recruit a Hindu group.

We also interviewed key informants who were selected to provide a range of perspectives from policy through to community physical activity promotion with an emphasis on physical activity for British minority ethnic groups. Key informants were suggested by the public health lead in NHS Health Scotland (see Table S2). Due to the active cross-sector working in physical activity in Scotland, this meant that we had access to a wide range of sectors including: sport and leisure services, policy makers, and community leaders. All key informants who were approached agreed to be interviewed.

### Ethics statement

The study was conducted with adherence to ethical guidelines for good practice in research [26] and ethical approval was granted by the Department of Applied Social Science (University of Stirling) Ethics Committee. Information sheets were sent to gatekeepers in advance of the focus groups and they were given an opportunity to ask questions about the process. They then circulated information sheets about the study to their groups. Information about the project was provided in English, Urdu and Punjabi. To support potential participants to make decisions about whether to participate, an independent person was available to talk through decisions in appropriate languages. At the beginning of the focus group participants were given the opportunity to ask questions and it was stressed that participation was voluntary and that they could withdraw at any time without giving a reason. Consent forms (available in the three languages) were then distributed and completed, with attention paid to ensuring that all participants were able to read and complete the forms. Given the potential language issues particular care was taken to ensure that participants clearly understood the nature of their involvement in the study and that confidentiality and anonymity would be maintained.

### Data generation

The focus groups were held at a time and place that was convenient to participants. Where possible, focus groups were held in the venue where groups were accustomed to meet. Groups were convened in community centres, apart from a prayer group that met in a large city centre café. In order to increase participation we offered child care expenses (of up to £20) and gave an honorarium of £20 (in high street vouchers) to cover any travel costs and inconvenience caused by attending the session. All but three of the focus groups were conducted in English; in the other three, both Punjabi and English were spoken, with roughly 50% of the participants speaking Punjabi. Interviews with key informants were conducted in English either face to face or over the telephone. Topic guides were used for both the focus groups and the key informant interviews (Appendices S1 and S2). The topic guides were developed by members of the research team, two of whom come from South Asian backgrounds. They also drew on previous research and were designed to answer the study questions. Data collection followed an iterative approach, responding to emerging themes and following up new lines of enquiry as appropriate.

### Data handling and analysis

The interviews and focus group discussions were digitally recorded, transcribed, and translated (where necessary) by GA and analysis was facilitated by use of NVivo 7. Data saturation was reached by the final focus group. We undertook a thematic analysis, using both inductive and deductive coding [27], exploring themes in relation to categories of ‘difference’ such as social class, age of parents and children, sex, migration history, geographical location (urban/rural) and country of origin [28]. We were also mindful of the differences between the terms ‘physical activity’ and ‘exercise’ which can describe different concepts [29]. Constant comparison (checking experiences against those of others in the sample) ensured that the thematic analysis represented all perspectives and negative cases were sought [30]. Analysis also included unanticipated themes [28]. In addition, we ensured that we did not ignore the nature of diversity (‘difference’) and variation of experience and perspective within the South Asian communities.

The researchers in the team came from a range of disciplines including medicine, nursing, sociology, psychology and social anthropology. Two members of the team were of South Asian origin. The range of disciplines and backgrounds enabled us to reflect on the data from a number of perspectives. However, we were also mindful of the influences that the researchers and the research team bring to the research process (reflexivity) [31]. The researchers who carried out the interviews and focus groups were both women (GA and FH). Neither had a strong interest in physical activity, which could have influenced the data collection. The analysis was carried out by FH, GA and RJ who compared and discussed the themes to ensure that bias was minimised at the analysis stage. The themes were also discussed amongst the wider research team. As is common in qualitative analysis, rigour was not established by double coding, but by members of the analysis team sharing and discussing transcripts and benefiting from a range of perspectives [32]. FH did all initial coding on the focus groups and GA was responsible for the key informant interviews. RH read through all transcripts and co-ordinated the developing coding frames. The analysis followed an interpretive approach commonly used in the social sciences [33], which is informed by theoretical insights from the beginning of the research (in this case theories of ethnicity and other forms of ‘difference’) and retains a reflexive stance to ensure that bias is minimised.

## Results

A total of 59 people took part in nine focus groups. Table S1 summarises the composition of these groups, indicating amongst other things their respective countries of origin. Our sample reflects the fact that the largest number of South Asians living in Scotland is Pakistani Muslims [34]. Table S2 illustrates the sample of 10 key informants who were interviewed. All of those who were approached agreed to be interviewed.

As we mentioned previously, although we asked about barriers to physical activity, we do not report them in this paper. In summary, they were very similar to those reported in other papers, bit for people from BME communities (e.g. fear of racism, single sex facilities) or the general population (e.g. cost, child care, weather). It is important to note that we uncovered a range of opinions, so that what may represent a motivator for some was not seen as such by others. For this reason, where data allow, we aim to provide a balanced and nuanced picture that deliberately seeks to avoid making the kinds of sweeping statements that lead to inappropriate stereotypes with a strong emphasis on cultural difference. As a recent critique of ‘cultural competency’ argues, we should be mindful of the variation of experience within minority ethnic communities and be wary of rendering such a complex notion such as ‘culture’ into something fixed and immutable [35]. Where data allowed, we also paid attention to variations linked to gender, age and degree of affluence or deprivation. We tried to differentiate between motivators and facilitators which were similar to those of the ‘majority population’ and those which were specific to people of South Asian origin. Analysis did not focus on their roles as parents, but took this into considerations when developing themes. A selection of quotes is used in the paper to illustrate the themes; further quotes are available from the corresponding author on request. Box S1 summarises the main themes emerging from the analysis.

### Types of physical activity people engaged in

Similar to the majority UK population, participants reported engaging in a wide range of physical activities and there was a great deal of variation in levels of physical activity between individuals. Many participants talked about enjoying (or wanting to participate in) walking, swimming and going to the gym (for primarily intrinsic motivation factors), which are similar activities that the UK majority population enjoy [36]. Activities that participants engaged in differed by gender; many of the male participants worked in shops or businesses and spoke about the physical activity gained through work, although there was some variation in opinion around this. While some regarded their working day as very active because of tasks such as shelf-stacking and the lifting and carrying of heavy boxes, others appeared to view their work as predominantly sedentary, possibly related to the presence or absence of shop assistants.

*M2: “But I mean when you are working… I mean when I was working as a shopkeeper or a newsagent or whatever I was doing a lot of physical work. I mean standing up there behind the counter for 10/12 hours, lifting heavy things, going to the cash and carry and carrying things, so you do get exercise like that.”*

*(Focus Group 7, Pakistani men)*

Football was an activity that was particularly enjoyed (or would be enjoyed if they had the opportunity), although one participant (M3) seemed less enthusiastic.

*GA: “What type of exercise or activity would you most like to take part in?”*

*M3: “Kabaddi “ [team sport which is played in India, Pakistan and Bangladesh]”*

*GA: “Really?”*

*M1: “I just want to play five a side game of football.”*

*M4: “All members would take part in that, I would guarantee that”*

*M3: “They don't pass you the ball and you get fed up with that.”*

*M1: “Just say you can't get the ball because you can't run after the ball. We would like to take part in anything that involves a few bodies…”*

*M4: “So we get a laugh…”*

*M1: “Yeah, so we get a laugh”*

*(Focus Group 5, Indian men)*

Men also mentioned enjoying social activities including cricket, walking (predominantly within the confines of their local neighbourhood and with friends), badminton and going to the gym. Walking in the countryside and swimming were mentioned less frequently.

Women in all of the groups talked of going to the gym (or using indoor gym equipment), taking part in team sports (e.g. netball), and participating in exercise classes. However, swimming and walking were the two activities that stood out as being the ones that women engaged in (or wished to engage in). Walking in particular was something that many felt that they could incorporate into their busy lives.

*F1: “…And I do a lot of walking as well.”*

*FH: “ where do you go walking?”*

*F1: ”Just up and down, you know, just going to the shops and that.”*

*FH: “Right.”*

*F1: “So just make it a regular part of my day, do a bit of shopping and stuff. So I just kind of try and do that, and also housework as well, try and do stuff like that.”*

*(Focus Group 4, Bangladeshi women)*

Most participants reported that their children took part in school-based activities or clubs, but physical activity for children outside school appeared to vary. The most frequently reported activity for children was swimming (particularly younger children), football, being out on their bikes (usually in the local area close to home), or playing outside with their friends. However some spoke of their fear of letting their children play outside because of safety issues. The participants also spoke about how their child's participation in non-school based physical activity often declined once they reached the teenage years.

From the discussions, many families appeared to do little physical activity together, partly owing to the work commitments of the men. Working in shops or as taxi drivers, for example, meant that they rarely had time off at weekends to spend with their wives and children. Several men also commented on the significant amount of time that they spent at their religious centre (mosque or temple); those that did take exercise as a family talked about activities such as badminton and walking.

There was no single physical activity that participants talked about consistently as not wanting to participate in. However, some activities, such as rugby and lawn bowls were mentioned by some individuals as particular activities which they did not feel were relevant to them or their communities. Both positive and negative attitudes towards engaging in activities in the countryside (e.g. hill walking, rock-climbing) were expressed and these differed between the groups.

### Social interaction

Social interaction and enjoyment were the key motivators for participants. There was a preference for taking part in physical activity with one or more friends rather than going along to a group session alone or exercising alone. This was common to both men and women, regardless of ethnic, religious or linguistic group. This was illustrated by the discussions between a group of men who were active in sport on the factors which motivated them:

*GA: “What are the key things that motivate you to do that [be physically active], what is it?”*

*M3: “For me it is just getting together with these guys, that's my…”*

*GA: “Social thing?”*

*M3: “Yeah because we get to see the guys twice a week or three times a week for a match or training.”*

*M2: “That's so nice.”*

*M3: “It is the social aspect you know, like you can get your brothers [used in religious sense] and playing competitively and going to training and going to the gym and stuff for me that's the main thing.”*

*(Focus Group 6, Pakistani men)*

Women also talked about how they enjoyed the social interaction

*FH: “So is it a kind of social… do you like the idea of doing activities together?”*

*F2: “Yeah, it's more fun as well.”*

*F3: “It motivates you more than doing it on your own doesn't it?”*

*F2: “Yeah, definitely, definitely. So you'll want to go out more if you know your friends are coming as well, you know, it's much better.”*

*(Focus Group 4, Bangladeshi women)*

In fact, the social aspect to engaging in physical activity was so important that some women felt that they would not do it on their own:

*F1: “I don't know about you guys but it has to be a team thing, not [something I want to do] on my own.”*

*F2: “The aerobics was an organised event and we all went, that's what we were doing in the past.”*

*GA: “Basically you would go if there were other people with you?”*

*F7: “If there are a few people going at the same time then that is good, 2 or three*

*friends”.*

*(Focus group 1, Pakistani women)*

Team sports (e.g. football and netball), badminton, swimming, and dance or exercise classes all offered the opportunity for social interaction. Walking groups were also mentioned by some groups.

Religious places (e.g. the temple or the mosque) featured strongly in the research as a place where people met in a group situation (particularly for the men). For some, it was the main place that they went to in the week and they could spend a considerable amount of time there. One man suggested that they could take the lead in encouraging people to take part in physical activity,

*M4: “Even if it was, even if there was some sports facility through the mosque if not the communities, the mosque should be able to provide some sort of, the mosque, this lesson can be preached through the mosques, if you want, you know, so that they are aware that this is an issue they can address it back in their own groups.”*

*(Focus Group 8, Pakistani mixed)*

### Enjoyment of exercise

Enjoyment of exercise was commonly mentioned as a motivator for both men and women especially when it included a social element. Dance was seen as one form of exercise that provided both “fun” and social interaction. One key informant thought that it was a significant form of physical activity for females in his community:

*“I know that a lot of the younger women, any time they've mentioned Bhangra [Indian dancing] aerobics they've been like yeah, we'll do that. Anything that's got a bit of a twist to it that adds maybe an element of their culture into it they're probably prone to go ahead and do. Like just even with dancing. I mean a form of physical activity for me is…a lot of girls I know on the weekends they'll get together and they'll have Bollywood music on one of the channels and they'll master some of the moves and they'll do that. Well that's exercise. That's physical activity. The only thing is you don't realise it is because you're enjoying it so much. You enjoy doing something and it's not a chore.”*

*(Key Informant 9, male)*

While some women favoured dancing to Bollywood videos or taking part in Bhangra dancing, one woman raised religious concerns:

*F3: “I don't like doing that [Bhangra dancing] to music because it is not allowed in Islam, Indian music and songs. I don't want me or my family to participate in that, you know.“*

*(Focus Group 8, Pakistani men and women)*

Since Bhangra dancing is related to the Sikh religion and culture, this particular participant felt it was inappropriate for Muslims. However, one of the women only focus groups, who were the most conservatively dressed in terms of modest clothing and the wearing of ‘Hijab’ (the Muslim women's head covering), expressed strong preferences for Bollywood dancing.

*GA: “So what would make it easier to get some kind of exercise into your life?”*

*F1: “An attraction. Like I said there is a Bollywood video. It would something that*

*would be light hearted, not too serious.”*

*F4: “Something that is a laugh as well and you can exercise, like a two in one purpose.”*

*(Focus Group 1, Pakistani women)*

Although women noted that this was not really permitted by Islam, they felt that it was acceptable in the privacy of their own homes with friends.

Men also described the importance of enjoyment in physical activity

*M3: “I think fun; fun is the main thing for me. If you didn't have fun doing something what's the point in doing it…Do you know what I mean? And I think when we play, we play like a lot of 5 a sides and 11 a side and I will be honest I know we all like to win and that, but sometimes when you lose I don't mind and I know that's bad, I think that's a personal thing that's bad but I sometimes have more fun because knowing that I have got, like everyone said, a kind of social group, a network, people you can talk to and not talk to and yet you know when we play tennis we are not very good at tennis for example but we have fun, you know. You feel….”*

*(Focus Group 6, Pakistani men)*

### Mental and physical benefits

The perceived mental and physical benefits of physical activity were mentioned as a motivator, but not as often as the social and enjoyment motivators (and not necessarily linked with them). Losing weight and increased fitness was mentioned by women in one group but not in the other groups:

*GA: “What motivates you to exercise?”*

*F1: “Well we look at slimmer people makes us really motivated (starts laughing).”*

*F2: “General fitness as well because you find yourself getting better.”*

*(Focus Group 1, Pakistani women)*

Several people, both participants in the focus group and the key informants talked about the increase in self-esteem associated with participating in physical activity. One key informant who ran outdoor activities for South Asian women reported that after taking part in one of her courses it was wonderful to see how some of the women became much more confident in general so that the skills that they learnt for exercising in the outdoors somehow translated into higher self-esteem and a greater confidence in general. One key informant also mentioned the increase in self-esteem she noticed after women had been taking part in exercise classes:

*“They found something where they can come and enjoy, get some exercise done and they have started taking an interest in themselves, valuing themselves, that “I'm worth something. And I respect my body and I respect myself.” So self respect has started to generate now.”*

*(Key informant 10, female)*

### Leadership and role models

Several people talked about how they would appreciate having someone to lead activities and be a motivator, such as having someone from their own community organising activities. One key informant also felt that religious centres could take on a leadership role in promoting physical activity:

*“And I sometimes think even religious groups in terms of the work I do, if religious institutions could encourage it and in partnership with what they teach, because active… being fit and something is not discouraged at all, no religion says it's bad, it's encouraged because essentially it gives you a better and healthier life.”*

*(Key informant 9, male)*

Some group participants and key informants also spoke about the importance of role models such as sport personalities (e.g. boxers). However, several people commented that there were not many role models around for them. Although well-known people were mentioned as role models (including Bollywood actors), participants also talked about having people in their local communities or families as role models. For example, one key informant noted that by going out running herself, other women could say, “*oh, you're doing it, I could possibly be doing it*” (Key Informant 8, female). Similarly one group of women talked about seeing a woman from South Asia running the marathon:

*F1: “.because I saw somebody in the London Marathon just on telly, obviously a Muslim woman, and she had a headscarf on but she was running a marathon, so I thought oh, so she must have something very light or something. She was wearing tracksuit trousers, full length, and she had full length shirt on and hair was covered because of her scarf.”*

*F2: “It's good to see that on TV as well actually, because it might encourage other ladies to think, well if she can do it then we certainly can as well.”*

*(Focus Group 4, Bangladeshi women)*

One female key informant had consulted community leaders about sports that children would want to participate in and cricket was reported to be an important sport for them:

*“We did a bit of consultation with some of the community leaders and that [cricket] is something that had a high value, high status. A lot of the young people's role models were famous cricketers from India, Pakistan and South Asia generally and that was something a lot of the young people aspired to be successful at.”*

*(Key Informant 6, female)*

She also said that some of the children were not necessarily fans of British sports so their role models were from South Asian countries rather than the UK. However, it is possibly something that all first or second generation migrants to the UK experience – for example, Americans may prefer basketball or baseball, and their roles models could be baseball stars.

Although sports personalities were seen as people to emulate and admire, one person commented on the fact that it was “*their profession to stay fit*” (Pakistani male) possibly implying that they were less inspirational because they were paid to do it as their job.

## Discussion

### Statement of principal findings

We found that South Asian-origin adults living in Scotland view physical activity in a broadly similar way to the general population. Many of the reasons that participants gave for what motivated them to participate in physical activity – perceived health benefits, sense of achievement and enjoyment – were similar to the majority population [37], [38]. However, there may be important, subtle cultural differences in the ways that people from South Asia socialise, or what activities they find enjoyable. For example, religion and the centrality of cross-generational family relationships played particularly important roles in how many people socialised (particularly those with young families). Therefore, these contexts may need to be taken into consideration when developing services or interventions. Visible role models were also viewed as important at both a national (e.g. well known sports personalities) and local (community members or lay members engaging in sports such as the marathon). Intrinsic motivations for participating in sport or physical activity were less commonly mentioned.

### Strengths and weaknesses of this study

The complexities of carrying out research with ethnic minority groups and faith groups are well known, with particular challenges around recruitment [15], [39]. We recruited women and men from a range of religious backgrounds, some of whom spoke little or no English, resulting in data which was rich in description of their views of physical activity. Having a researcher who was South Asian and well linked into many relevant networks ensured that we were able to access a wide range of participants. Due to the diverse methods of recruitment (e.g. through a radio interview, using community leaders, advertising) it is not possible to provide details of how many people were approached to take part, and how many declined. Carefully facilitated focus groups undertaken in places and in languages in which participants were comfortable facilitated open and relaxed discussions. The inclusion of key informants who were highly involved in service provision complemented and enriched the focus group derived data. Purposive sampling of participants who were both inactive and active, and from a range of different economic, religious and linguistic groups proved effective in enabling us to achieve saturation on the key themes. Having a multidisciplinary group of researchers enabled the analysis and conduct of the study to be carried out with insights into both medical and socio-cultural perspectives.

One limitation is that it is difficult in qualitative studies of this kind to be certain of the broader generalisability of our findings. However, we believe that the depth of enquiry has generated insights that are likely to be broadly transferable to South Asians minority populations living in other parts of the world. A further possible limitation was that the research was commissioned by NHS Health Scotland to answer specific questions. Whilst this is a strength in that it made the study highly relevant to policy and service development, it may also be viewed as a limitation as the researchers were somewhat restricted in the breadth of issues they could explore. Finally, we decided, where possible to focus on pre-existing groups to facilitate recruitment. Whilst this may lessen the time needed for people to feel comfortable with each other, facilitation of the discussion may have been constrained by pre-existing group dynamics.

### Contextualising this work within the wider literature

Although it has been well over a decade since many of the barriers to exercise were first identified, there remain major structural barriers to South Asians taking part in regular exercise. This study found that South Asians living in Scotland view physical activity in a similar way to the general population, have similar motivations and they enjoy (or would like to enjoy) more or less the same activities (particularly swimming, walking and using the gym).

Undertaking physical activity for the opportunity for enjoyment and the social opportunities it provides has been identified as a significant motivator in a number of studies for the general population [38], [40], [41]. However, there may be age differences, with younger people more likely to report these as motivators than older people [42].

Whilst some of the barriers we identified in our wider study were also similar to the majority population (cost, childcare and lack of time or motivation) they also face barriers which can severely restrict choice, particularly for women. For example, swimming and using the gym are two of the most popular activities for everyone in the UK including South Asians, but many South Asian women are unable to use their local leisure centre because of culturally inappropriate facilities. Many South Asian people (particularly women) are thus effectively denied access to some of the leisure services. There have been a number of successful projects designed specifically for South Asians (e.g. Khush Dil [43] and Project Dil [44]) which have addressed these barriers.

The barriers outlined above impacted not only on the adults we interviewed, but also the type and amount of physical activity that their children participated in. Evidence from research suggests that South Asian and British minority ethnic children tend to exercise less than the general population [8]–[10]. The barriers we identified also meant that it was difficult for parents (particularly mothers) to exercise with their children and provide a range of physical activity opportunities for them.

### Implications for policy, practice and research

Strategies for encouraging South Asians to take part in a range of physical activities need recognise that motivations focus on the enjoyment and socialisation opportunities, and only to a lesser extent on health benefits and the activity itself. Focus should be placed on group based activities as South Asians often have strong community, religious, work or family ties, which makes it difficult to do physical activity as individuals.

Role models can help motivate and inspire participation in a broader range of activities. The motivations we have identified in this study need to be placed alongside the identified barriers in order to develop and deliver effective services and services. From our wider study we found that, for those of our participants who were non-active, there were significant barriers that made it difficult to overcome the first hurdle to becoming active. Although many of the barriers to taking part in physical activity are similar to the majority population (e.g. lack of time, child care facilities), South Asian communities still face additional barriers such as the lack of women only facilities or activities. Many of our participants stressed that they didn't necessarily need facilities exclusive for South Asian women, but they did require strictly women-only spaces/facilities/opportunities. In addition the context of the lives of South Asian (such as the places and ways in which they socialise) need to be considered to maximise the uptake and acceptability of such services. We have alluded to institutional racism via inappropriate leisure facilities, but fear of personal racism and safety issues also affected the choices that families made for their children, or restricted the choices that women made for themselves. For example, some women were reluctant to go out jogging because of the negative attention they attracted. Box S2 provides some explanations and implications for clinicians and policy makers.

Interestingly the motivators that participants described were the same for different religious groups and between genders, suggesting that interventions that focus on motivators, although they need to take account of barriers, may have common underlying components.

### Conclusions and Recommendations

UK South Asians are generally substantially less physically active than the UK general population which makes a significant contribution to their levels of diseases such as diabetes and heart disease. Research and strategies often focus on the barriers to participation rather than the motivators and facilitators. This study has identified several motivators such as socialisation and enjoyment which could increase participation. Role models could also act as facilitators. Strategies and interventions should recognise the importance of these motivators and facilitators alongside the recognised barriers.

## Supporting Information

Table S1 **Details of focus groups.**
(DOCX)

Table S2 **Details of Key Informants.**
(DOCX)

Box S1 **Main themes emerging from the data.**
(DOCX)

Box S2 **Explanations and implications for clinicians and policymakers.**
(DOCX)

Appendix S1 **Topic guide for South Asian focus groups.**
(DOCX)

Appendix S2 **Interview topic guide for physical activity specialist and community group leaders.**
(DOCX)
