# Supplementary material for: Physical Activity in South Asians: An In-Depth Qualitative Study to Explore Motivations and Facilitators
Source: PLoS One. 2012 Oct 10;7(10):e45333. doi: 10.1371/journal.pone.0045333 (PMC3468573; doi:10.1371/journal.pone.0045333)
Supplement: Table S1 — Details of focus groups. (DOCX) [file pone.0045333.s001.docx]

**Table S1: Details of focus groups**

| ***ID*** | ***Location*** | ***Number of participants*** | ***Country of origin*** | ***Religion*** | ***Affluent / disadvantage*** | ***Gender*** | ***Active / non-active*** |
| --- | --- | --- | --- | --- | --- | --- | --- |
| FG1 | Aberdeen | 7 | Pakistan | Islam | Mainly affluent | Female | Non-active |
| FG2 | Edinburgh | 6 | Pakistan | Islam | Mainly affluent | Female | Mixed |
| FG3 | Glasgow | 4 | Pakistan | Islam | Mostly disadvantaged | Female | Active |
| FG4 | Aberdeen | 6 | Bangladesh | Islam | Mostly disadvantaged | Female | Non-active |
| FG5 | Glasgow | 7 | India | Sikh | Mostly disadvantaged | Male | Mixed |
| FG6 | Glasgow | 7 | Pakistan | Islam | Mainly affluent | Male | Active |
| FG7 | Edinburgh | 6 | Pakistan | Islam | Mostly disadvantaged | Male | Non-active |
| FG8 | Edinburgh | 6 | Pakistan | Islam | Mostly disadvantaged | Mixed | Mostly non-active |
| FG9 | Edinburgh | 10 | India | Sikh | Mostly affluent | Female | Mostly non-active |
