# Supplementary material for: Physical Activity in South Asians: An In-Depth Qualitative Study to Explore Motivations and Facilitators
Source: PLoS One. 2012 Oct 10;7(10):e45333. doi: 10.1371/journal.pone.0045333 (PMC3468573; doi:10.1371/journal.pone.0045333)
Supplement: Table S2 — Details of Key Informants. (DOCX) [file pone.0045333.s002.docx]

**Table S2: Details of Key Informants**

| *ID.* | *Role* | *Gender* | *Ethnicity* |
| --- | --- | --- | --- |
| Key Informant 1 | Leads outdoor education programmes | Female | Indian |
| Key Informant 2 | Policy role in promoting walking | Male | White |
| Key Informant 3 | Policy/promotion of active travel | Female | White |
| Key Informant 4 | Equalities remit within leisure centres | Female | Pakistani |
| Key Informant 5 | Physical activity and health policy role | Female | White |
| Key Informant 6 | Health policy role | Male | White |
| Key Informant 7 | Community development work | Female | Pakistani |
| Key Informant 8 | British minority ethnic women’s activity project | Female | Indian |
| Key Informant 9 | Community sports inclusion/equality remit | Male | Pakistani |
| Key Informant 10 | Yoga teaching/promotion | Female | Indian |
