# Supplementary material for: Physical Activity in South Asians: An In-Depth Qualitative Study to Explore Motivations and Facilitators
Source: PLoS One. 2012 Oct 10;7(10):e45333. doi: 10.1371/journal.pone.0045333 (PMC3468573; doi:10.1371/journal.pone.0045333)
Supplement: Box S1 — Main themes emerging from the data. (DOCX) [file pone.0045333.s003.docx]

**Box S1: Main themes emerging from the data**

**Types of activities South Asians mainly participated in**

- Men preferred football, the gym and cricket
- Women preferred walking, swimming and the gym
- Children most likely to participate in swimming activities, and playing outside
- Many families appeared to do little physical activity together

**Motivators to physical activity**

- Motivators were almost exclusively external (for a purpose other than physical activity) rather than internal (for its own sake)
- Motivators included fun, enjoyment and socialising and health benefits
- People more likely to be involved in physical activity that included a social aspect
- Role models were seen as important
