# Supplementary material for: Physical Activity in South Asians: An In-Depth Qualitative Study to Explore Motivations and Facilitators
Source: PLoS One. 2012 Oct 10;7(10):e45333. doi: 10.1371/journal.pone.0045333 (PMC3468573; doi:10.1371/journal.pone.0045333)
Supplement: Box S2 — Explanations and implications for clinicians and policymakers. (DOCX) [file pone.0045333.s004.docx]

**Box S2. Explanations and implications for clinicians and policymakers**

- Promoting physical activity solely for its health benefits is unlikely to be a success for South Asian populations.
- Physical activity interventions are most likely to succeed if they include the opportunity for social engagement or enjoyment.

The following should be considered when recommending or developing strategies to increase physical activity:

- Working in partnership with religious centres and community organisations to provide physical activity opportunities or information. Creating opportunities to exercise in a familiar setting such as a religious or community centre.
- Drawing on role models from South Asian backgrounds (at a local and national level) to provide inspiration and motivation
- Introduce people to a range of physical activities that they may enjoy via the provision of taster sessions
- Focus on physical activity opportunities for family units and other groups (e.g. football, netball, dance classes)
- Address the known barriers to physical activity in South Asians such as lack of women only activities, and safety issues
