# Supplementary material for: Physical Activity in South Asians: An In-Depth Qualitative Study to Explore Motivations and Facilitators
Source: PLoS One. 2012 Oct 10;7(10):e45333. doi: 10.1371/journal.pone.0045333 (PMC3468573; doi:10.1371/journal.pone.0045333)
Supplement: Appendix S1 — Topic guide for South Asian focus groups. (DOCX) [file pone.0045333.s005.docx]

**APPENDIX S1**

**Topic guide for South Asian focus groups**

**Introductions**

- About the project
- Researchers and participant introductions
- Consent & demographic forms

1. **Defining physical activity and exercise**

What do you think of as physical activity and exercise?

- - Competitive or team sport e.g. team or individual athletics, cricket, badminton, squash
  - Activities for leisure e.g. walking, swimming, cycling
  - Others kinds of activity: dog-walking; taking kids to the park; gardening; dancing, manual labour, housework

1. **General attitudes towards physical activity and exercise.**

We would like to know a bit about what kind of physical activity you do at the moment and what your general thoughts are about taking exercise. First of all:

Physical activity is actually anything that uses up energy, while exercise is about things like playing tennis, swimming and so on. Bearing this in mind, how does physical activity fit in with your daily life at the moment?

- What is a typical day like? (use of car or walking; active job or home life etc; amount of non-active things like TV watching / reading / listening to music)
- Emotional energy used up dealing with family etc?

How do you feel about taking exercise in general?

(Exploring their personal attitudes to exercise rather than their knowledge about exercise for health)

- Involved in exercise at the moment?
- What kinds? (Aerobics at home / class; team sports, etc)

**3. Barriers and motivators to exercise & physical activity**

What kinds of exercise/physical activity would you be most likely to do or take part in? Why?

What kinds of exercise/physical activity would you be least likely to do or take part in? Why?

For those of you who are not involved in taking much exercise, what do you think the main things are that make it difficult for you to be more active?

- On a personal level: Having very small children, tiredness, ill health, caring for relatives, too busy at work / work long hours, no-one to go to the exercise class/gym with?
- On the family level: How do friends / relatives / partners view exercise? Any differences for men or women?
- Are there any religious or cultural factors?, language issues, not knowing where to go, not feeling comfortable going to a place where there are no members of your community, fear of and/or experience of being treated unfairly
- Institutional level: do leisure centres provide women only sessions etc; clothing – is this restrictive and is there sportswear available that suits their needs / culture etc

What would make it easier to fit some kind of exercise or just more physical activity into your daily life?

What sort of activities would you enjoy and what do you think is just not appropriate for you?

- cultural/religious appropriateness, local facilities, feeling safe, exercise instructors who can speak my language, culturally specific exercise bhangra dancing, cost, more understanding of weight and exercise issues, fitness assessments, exercise consultations; help with childcare

For those of you who regularly take exercise – how do you manage to fit it in with your daily life?

- What motivates you? (e.g. Enjoyment of the activity itself, getting out of the house, exercising with friends, health benefits like weight loss etc)
- Did you have any obstacles to overcome before you got into the habit of taking regular exercise? (e.g. Inappropriate facilities, racism, cost, access, lack of time or childcare, attitude of partner or relatives etc)

**4. Children and physical activity**

What kind activities are your children involved in on a day to day basis that you think can be defined as exercise and as physical activities (prompt: walking to school, at school, after school activities, weekends, **is there a difference between boy children and girl children and different age groups**)

How do you feel about this?

- Do they watch much TV, use computer games, DVDs etc?
- When and where are they most active: at home, school, outside with friends, outside with family, in the gym or swimming pool?
- Do you encourage them to be active / more active or not (explore why/why not)?

Is there anything that stops your children from taking part in physical activity and exercise? (Prompt: bullying, fear of safety of children and as above. **Also explore issues for girl children and boy children and different age groups**)

**5. Family and physical activity**

What do you enjoy doing as a family? How do you normally spend your time together?

What sort of things do you do as a family that are physically active? (with or without partners)

- Going for walks
- Playing with the kids at home / park / swimming pool / leisure centre
- What do you most enjoy doing together?
- What would encourage you all to be more active as a family?
- What stops you from being active as a family?

**6. Attitudes to the ‘outdoors’ v indoor centres for exercise**

How do you feel about going out into the countryside for walks compared with doing exercise in a gym or leisure centre?

- How do you feel about the ‘outdoors’ in general? (would they ever take the kids camping, hillwalking etc?).
- What does the term ‘outdoors’ mean to them?
- Is there a particular place in the outdoors that you enjoy (e.g. beach, river, hills)
- How do you feel going into a gym (e.g. a pulse centre with exercise machines and weights) or the swimming pool

**7. Physical activity and health**

When I said a little bit about why NHS Scotland asked us to do this research, I mentioned the link between heart disease, diabetes and physical activity. Was this something that you were already aware of?

- Does knowing about the health benefits have any impact on your likelihood of taking up or taking part in exercise or simply becoming more active?

**8. What physical activity facilities or services they would like (can suggest anything, think laterally)**

If you had a say in designing services or facilities to encourage physical activity or sport, what would you want for you, your family or your children?

- e.g. women only sessions at the gym, walking groups for Muslim women, more information about relevant services, childcare facilities, workout videos that are culturally specific, free services, more access to services, different opening hours.
