# Supplementary material for: Physical Activity in South Asians: An In-Depth Qualitative Study to Explore Motivations and Facilitators
Source: PLoS One. 2012 Oct 10;7(10):e45333. doi: 10.1371/journal.pone.0045333 (PMC3468573; doi:10.1371/journal.pone.0045333)
Supplement: Appendix S2 — Interview topic guide for physical activity specialist and community group leaders. (DOCX) [file pone.0045333.s006.docx]

**APPENDIX S2**

**Interview topic guide for physical activity specialist and community group leaders**

1. **Defining physical activity and exercise**

What do you think of as physical activity and exercise?

- - Competitive or team sport e.g. team or individual athletics, cricket, badminton, squash
  - Activities for leisure e.g. walking, swimming, cycling
  - Others kinds of activity: dog-walking; taking kids to the park; gardening; dancing, manual labour, housework

1. **General attitudes of their community group/population towards physical activity and exercise.**

What do you think people in your community think and know about physical activity, and exercise?

How do they view it in relation to health?

What kinds of physical activities are they generally involved with?

**3. Barriers and motivators to exercise & physical activity**

What kinds of exercise/physical activity would your community group/population be most likely to take part in/do (explore why)?

What kinds of exercise/physical activity would your community group/population be least likely to take part in/do (explore why)?

What do you think the main things are that make it difficult for your community group/population to be more active?

- On a personal level: Having very small children, tiredness, ill health, caring for relatives, too busy at work / work long hours, no-one to go to the exercise class/gym with?
- Any differences for men or women?
- Religious and cultural factors, language issues, not knowing where to go, not feeling comfortable going to a place where there are no members of your community, fear of and/or experience of being treated unfairly
- Institutional level: do leisure centres provide women only sessions etc; clothing – is this restrictive and is there sportswear available that suits their needs / culture etc

What would make it easier to fit some kind of exercise or just more physical activity into your community group/population daily lives?

What sort of activities would your community group/population enjoy and what do you think is just not appropriate for them?

- cultural/religious appropriateness, local facilities, feeling safe, exercise instructors who can speak my language, culturally specific exercise bhangra dancing, cost, more understanding of weight and exercise issues, fitness assessments, exercise consultations; help with childcare

1. **Defining physical activity and exercise**

What do you think of as physical activity and exercise?

- - Competitive or team sport e.g. team or individual athletics, cricket, badminton, squash
  - Activities for leisure e.g. walking, swimming, cycling
  - Others kinds of activity: dog-walking; taking kids to the park; gardening; dancing, manual labour, housework

1. **General attitudes of their community group/population towards physical activity and exercise.**

What do you think people in your community think and know about physical activity, and exercise?

How do they view it in relation to health?

What kinds of physical activities are they generally involved with?

**3. Barriers and motivators to exercise & physical activity**

What kinds of exercise/physical activity would your community group/population be most likely to take part in/do (explore why)?

What kinds of exercise/physical activity would your community group/population be least likely to take part in/do (explore why)?

What do you think the main things are that make it difficult for your community group/population to be more active?

- On a personal level: Having very small children, tiredness, ill health, caring for relatives, too busy at work / work long hours, no-one to go to the exercise class/gym with?
- Any differences for men or women?
- Religious and cultural factors, language issues, not knowing where to go, not feeling comfortable going to a place where there are no members of your community, fear of and/or experience of being treated unfairly
- Institutional level: do leisure centres provide women only sessions etc; clothing – is this restrictive and is there sportswear available that suits their needs / culture etc

What would make it easier to fit some kind of exercise or just more physical activity into your community group/population daily lives?

What sort of activities would your community group/population enjoy and what do you think is just not appropriate for them?

- cultural/religious appropriateness, local facilities, feeling safe, exercise instructors who can speak my language, culturally specific exercise bhangra dancing, cost, more understanding of weight and exercise issues, fitness assessments, exercise consultations; help with childcare

**4. Children and physical activity**

What kind activities are your community group/population children involved in on a day to day basis that you think can be defined as exercise and as physical activities (prompt: walking to school, at school, after school activities, weekends, **is there a difference between boy children and girl children and different age groups**)

Is there anything that stops the children in your community/population from taking part in physical activity and exercise? (Prompt: bullying, fear of safety of children and as above. **Also explore issues for girl children and boy children and different age groups**)

**5. Attitudes to the ‘outdoors’ v indoor centres for exercise**

How do your community group/population feel about going out into the countryside for walks compared with doing exercise in a gym or leisure centre?

**6. Examples of services and interventions that have worked and ones that have not**

Are you aware of any services or interventions to encourage physical activity for your community/population?

Which or them were successful and why?

Which of them were unsuccessful and why?

**7. What physical activity facilities or services they would like (can suggest anything, think laterally)**

If you had a say in designing services or facilities to encourage physical activity or sport,what would you want for your community/population? e.g. women only sessions at the gym, walking groups for Muslim women, more information about relevant services, childcare facilities, workout videos that are culturally specific, free services, more access to services, different opening hours.
